# Toxicity test of a dental commercial composite

**DOI:** 10.4317/jced.52201

**Published:** 2015-04-01

**Authors:** Santa Ponce-Bravo, Constantino Ledesma-Montes, José-Luis Martínez-Rivera, Maricela Garcés-Ortíz

**Affiliations:** 1 Oral Pathology Laboratory. División de Estudios de Posgrado e Investigación. Facultad de Odontología, Universidad Nacional Autónoma de México. México, Distrito Federal. MÉXICO

## Abstract

**Background:**

International rules must be followed for testing biosecurity in dental materials. A new brand of restorative material appeared in the market and regulations indicated that it should be tested for toxicity.

**Objectives:**

The aim of this study was to determine the 90-day sub chronic toxicity of one triethylene glycol dimethacrylate containing composite (MEDENTAL Light-Cure Composite™) orally administered to rats according to Organization for Economic Co-Operation and Development no. 48 guidelines and the requirements specified in the ISO 10993-11.

**Material and Methods:**

Wistar rats ate the polymerized composite during 90 days and were observed to determine changes in their behavior, eye and skin signs and other attitudes such as aggressiveness, posture, walking and response to handling. After 90 days were sacrificed to ascertain blood alterations, we did special hematological tests and assessed microscopic slides from 33 different organs.

**Results:**

We recorded no significant changes in clinical behavior of the animals. Microscopic review of the H&E stained slides obtained from the analyzed organs showed no abnormal inflammatory or cytological changes and all hematological special tests were within normal limits.

**Conclusions:**

Results of this study show that under our experimental conditions the MEDENTAL Light-Cure Composite™ does not produce inflammatory or cytological changes suggestive of toxicity.

** Key words:**Dental materials, composite resin, toxicity, inflammation, TEGDMA.

## Introduction

Since several years ago, different kinds of esthetic dental materials exist in the dental market and some of them are the reinforced composites that are now considered as useful restorative dental materials. However, each one has advantages and disadvantages during their use as tooth restoration materials (1). Physical-chemical properties of these reinforced composites (hardness and compressive strength) are the same as conventional composites (2) and studies made on different kinds of dental composites showed that the most important difference among them are: their ability to release fluoride, chemical adherence to dentin and compressive strength (2,3). Composites are new materials used in Dentistry with better physical, chemical, biological, radiological and esthetic properties. However, they have some adverse properties as dimensional stress, contraction during polymerization and cellular damage caused by their components (4).

Recently, polyacid compounds have been added to the composites and currently, they have higher viscosity and strength resistance (3,5). Chemical composition of composites is variable and they are composed by one organic component bisphenol Aglycidyl methacrylate (Bis-GMA) or triethylene glycol dimethacrylate (TEGDMA) plus another inorganic component (glass, zirconium, barium, quartz and strontium) and a chemical bond (silano and polyacid groups). The above-mentioned materials provide reduced contraction properties in the finally polymerized composite (2-4). In addition, it is known that saliva has a deleterious effect decomposing these compounds (6).

Several studies have been published on TEGDMA adverse effects under different experimental and clinical conditions and different parameters were evaluated (7-19); also, some reviews were published (8,20).

It has been demonstrated that TEGDMA induces mitochondrial damage and oxidative stress in human gingival fibroblasts and it causes apoptosis in primary human gingival fibroblasts (7,9). Other study reported that this compound showed a significant enhancement of glucose consumption and lactate production inducing GSH depletion and stimulating G6PDH and GR activity (10).

Studying TEGDMA metabolic effects, Engelmann et al. showed it increased the phosphomonoester concentration and decreased the phosphodiesters, enhancing the phospholipid turnover. TEGDMA changed the metabolic state of cells indicated by the slight decrease of nucleoside triphosphates and increasing the ratio of nucleoside diphosphates to nucleoside triphosphates. The most remarkable effect of TEGDMA was a nearly complete decline of intracellular glutathione levels (11).

Kehe *et al.* reported that TEGDMA is toxic to pulmonary cells and pointed on the risk for pulmonary cell damage (12) and it significantly suppressed TNF-α secretion from THP-1 monocytes stimulated with bacterial lipopolysaccharide suggesting that this alteration may influence the biological response of tissues to material in an inflammatory intraoral environment (13).

TEGDMA is toxic to human gingival fibroblasts since it alters the mitochondrial dehydrogenase (MTT) and the lactate dehydrogenase (LDH) activities (14). In another report, Volk *et al.* reported that TEGDMA depletes intracellular GSH levels at low concentrations suggesting that decrease of GSH is an early reaction triggered prior to other cytotoxic alterations (15) and Geurtsen *et al.* (16) found that TEGDMA has a very high cytotoxicity potential tested in human primary fibroblast cultures. Wataha *et al.* concluded that TEGDMA containing composites affect or alter cellular functions in Balb/c 3T3 fibroblasts (20).

Schweikl *et al.* (17) recently reviewed the genetic toxicology of TEGDMA and Geurtsen and Leyhausen wrote on its chemical and biological interactions (8).

There are few reports on skin reactions associated to TEGDMA: Kanerva reported on the skin allergic reactions to TEGDMA in 8.9% of the patients tested (18) and Katsuno *et al.* demonstrated that three TEGDMA containing dentin-bonding systems caused contact dermatitis in the skin of guinea pigs (19). Although, the pathogenic mechanism is not well known (19,21).

In view of the above-mentioned results, we tested a composite commonly used in private and institutional dental practice in Mexico, the MEDENTAL Light-Cure Composite™. For this reason, the aim of this study was to know the 90-day oral toxicity of a TEGDMA containing dental composite (MEDENTAL Light-Cure Composite™) orally administered to rats according to OECD no. 48 guidelines and the requirements specified in the ISO 10993-11 (22,23).

## Material and Methods

Twenty Wistar rats, were used.

To perform the current study the recommendations of the OECD no. 48 guidelines and the requirements specified in the ISO 10993-11 were followed (22,23) and the protocol for this study was approved by the Ethics Committee of our institution. following the parameters of the above-mentioned documents recommending the use of 20 rats, we chose ten males and ten females, 9 week old, clinically healthy animals weighting 230-250 g.

TEGDMA containing composite (Light Cure Composite™, MEDENTAL Co., Mexico, Distrito Federal, Mexico) was prepared without the bonding system following the manufacturer instructions. The composite was polymerized with a photopolymerizing lamp during 40 sec, pulverized in a ceramic mortar and pestle and the pulverized powder was stored in sterile Eppendorf tubes. The rats were fed orally during 90 days with 100μg of the pulverized composite every day in the morning and then, they drank water and food pellets ad libitum. The rats were observed to determine changes in eye signs (irritation and inflammation), skin and mucous membranes (irritation, inflammation, secretions and pruritus), muck, urine (color, consistency, odor), behavior and autonomic activity (aggressiveness, posture, walk and response to handling) and other attitudes according to ISO 10993-11 regulations (23).

In day 90, the rats were anesthetized intra-muscularly with 2mL of acerpomazine and 1 mL/Kg of ketamine. Then, by intracardiac puncture, we obtained 5 mL of blood from each rat and stored in EDTA and anticoagulant containing tubes. Rats were sacrificed by aspiration of chloroform gas and 33 organs: cerebrum, cerebellum, spinal medulla (cervical, mid-thoracic and lumbar), pituitary, thyroid, parathyroid, thymus, esophagus, salivary glands, stomach, small intestine, large intestine, Peyer’s patches, liver, pancreas, kidney, adrenal, spleen, heart, trachea, lung, aorta, gonads (ovaries and testicles), prostate, urinary bladder, lymph nodes, peripheral nerves, bone marrow, skin and eyes were immediately immersed in 4% paraformaldehyde during 24 h.

Specimens were sent to the Clinical and Experimental Pathology Laboratory and routinely processed to obtain 3µm thick, paraffin embedded, H and E stained slides. All the slides were observed in a light transmitted microscope (Carl Zeiss, Germany) by two experienced Oral Pathologists.

The hematological studies were as follows: hematocrit, hemoglobin concentration, erythrocyte count, platelet count and total leukocyte count. Laboratory blood tests were glucose, urea, creatinine, cholesterol, alanine aminotransferase, alkaline phosphatase, total proteins, albumin, potassium and sodium blood-concentrations. These studies were carried out at the Laboratory of Pat-hology of the Facultad de Veterinaria y Zootecnia, UNAM. Normal animal values were according to the International Species Information System (24).

## Results

After 90 days, the rats gained the normal amount of weight. No alterations in the clinical eye signs, skin, mucous membranes, muck, urine, behavior (autonomic activity and attitude). During the microscopic analysis of the H&E stained slides, no cytological alterations were observed in any of the evaluated organs. Although, there were minor changes in the hematological tests, but all of them were always within normal limits.

## Discussion

Several studies on TEGDMA adverse effects have been published (7-19), demonstrating that TEGDMA induces different metabolic cellular effects as mitochondrial damage, producing depletion of the oxidative stress and apoptosis in human gingival fibroblasts altering mitochondrial dehydrogenase and lactate dehydrogenase activities (7,9,14). Results from other study, showed that it significantly enhanced glucose consumption and lactate production (10). In the Engelmann *et al.* study, TEGDMA increased the phosphomonoester concentration and decreased the phosphodiesters, enhancing the phospholipid turnover. Also, it slight decreased nucleoside triphosphates and increased the nucleoside diphosphates-nucleoside triphosphates ratio with a nearly complete decline of intracellular glutathione levels (11).

Volk *et al.* reported that TEGDMA depletes intracellular GSH levels at low concentrations suggesting that decrease of GSH suggesting it is an early cellular reaction prior to other cytotoxic alterations (15) and Wataha *et al.* concluded that TEGDMA affect Balb/c 3T3 fibroblasts cellular functions (20). The above mentioned results support the Geurtsen *et al.* (16) suggestion that TEGDMA has a very high cytotoxicity potential in human fibroblasts.

In 2001, Kehe *et al.* reported that TEGDMA may influence the biological response of tissues to material in an inflammatory intraoral environment suppressing TNF-α secretion from THP-1 monocytes (12) and Schweikl *et al.* (17) reviewed the genetic toxicology of TEGDMA. Additionally, there are few reports on skin reactions associated to TEGDMA (18,19,21). Although, the pathogenic mechanism is not well known (20,22).

These results prompted us to test if the commonly used restorative material MEDENTAL Light-Cure Composite™ produced clinical or microscopic changes.

During the study, we found no pathological changes in the evaluated parameters. Microscopic review of the slides from the 33 selected organs, evidence of inflammatory or pathologic cellular change was not found. Results of our study strongly suggest that ingestion of the well-polymerized composite we tested (MEDENTAL Light-Cure Composite™) did not produce clinical toxicity or behavioral or cytotoxic changes to the studied organs of the animals. Results of this study suggest that when the clinician uses this resin under the appropriate clinical conditions following the manufacturers’ instructions and the patient inadvertently ingests it, this composite is clinically safe. Under our experimental conditions it appears that, “non-polymerized components” of the tested composite did not exist or if they are produced, their presence during 90 days within the gastrointestinal apparatus and their absorption to the blood stream did not generate pathologic changes or systemic alterations in the studied animals.
